# Conducting Qualitative Interviews using Virtual Communication Tools amid COVID-19 Pandemic: A Learning Opportunity for Future Research

**DOI:** 10.31729/jnma.5738

**Published:** 2020-12-31

**Authors:** Lalita Kumari Sah, Devendra Raj Singh, Rajeeb Kumar Sah

**Affiliations:** 1 Faculty of Medicine Health and Social Care, Canterbury Christ Church University, Kent, United Kingdom; 2 Department of Public Health, Asian College for Advance Studies, Purbanchal University, Satdobato, Lalitpur, Nepal

**Keywords:** *COVID-19*, *fieldwork*, *qualitative interviews*, *virtual communication tools*

## Abstract

The COVID-19 pandemic has threatened the health and wellbeing of the global citizens which has led to a significant change in attitude, lifestyle and behaviour of people from diverse professions, including academics and researchers. Doctoral students and funded researchers with the time limit to their research project have experienced stress and anxiety due to the struggle of negotiating and managing timeline to complete the fieldwork for their research. In the current circumstances, increasing number of researchers are looking for alternative methods to conduct the fieldwork and complete the data collection. In this context, the aim of this viewpoint is to provide reflections on the challenges and opportunities experienced by the authors while conducting qualitative research fieldwork during the COVID-19 pandemic.

## INTRODUCTION

The COVID-19 pandemic has contributed to the wider structural changes in our society that has affected people's daily lives. For example, working from home is becoming normal and has possibly blurred the lines between personal and professional settings; travel restrictions has limited the movement of people around the world; national and local lockdowns have affected in-person individual or group meetings and social gatherings; and planning indoor and outdoor activities have become precarious and challenging. From researcher perspectives, this has constrained the planned and ongoing research activities both within and outside the health sectors.^1^ In this context, the viewpoint discusses the challenges and opportunities to use virtual communication tools while conducting qualitative research fieldwork during and beyond the pandemic.

## CHALLENGES TO QUALITATIVE RESEARCH FIELDWORK DURING COVID-19

Doctoral students and funded researchers with the time limit to their research project have experienced stress and anxiety due to the struggle of negotiating and managing timeline to complete the fieldwork for their research.^2^ Health related research where participants are recruited from the community or hospital settings faces extra obstacles due to the risk of community transmissions which is highly likely to be higher in such settings. Despite social distancing and other safety measures to protect participants and general public from the virus, the fear of the pandemic still exists within the community. Evidence suggests that many people who are COVID-19 positive may not show any symptoms but can still transmit the virus.^3^ In such situation, participants as well as researchers are hesitant to meet face-to-face or spend extended period of time in the field for the purpose of research interviews. Even if participants agree to take part in face-to-face interviews, they are likely to be anxious due to the fear of COVID-19 and may rush to end the interview sooner as they are likely to feel uncomfortable due to the current COVID-19 situation. Therefore, in the current circumstances, increasing number of researchers are looking for alternative methods to conduct the fieldwork and complete the data collection.

National and local lockdowns, social distancing and travel restrictions have forced universities and research institutions around the world to rethink their research strategy, especially in relation to the fieldwork, to safeguard and support both researchers and participants from COVID-19. Some institutions are encouraging researchers to complete the data collection using virtual communication tools while others argue that it may not be the best option to do so because of the challenges and disadvantages associated with the virtual interviewing. Doctoral students and funded researchers with the time limit to their research project are at the crossroads of taking quick decisions about the fieldwork for their project. Although many UK institutions and funding organisations have extended the deadline by 3 to 6 months with financial support,^4,5^ the anxiety of the researchers have not diminished as the pandemic continues. The decision-making for researchers who are required to travel to other countries or within the country to complete their fieldwork is further complicated as we continue to live and work under local and national restrictions. Moreover, the increase in travel costs, lack of regular flights and the requirements of self-isolation, in many cases on both sides, have presented additional challenges for such researchers. As a result, researchers experience issues of financial burden, time constraints, and other psychological concerns related to the fear of COVID-19 infection, possibility of lack of productivity and time management in the fieldwork. The experience of second wave of COVID-19 and uncertainties about returning to normal routine demands an immediate action to look for alternative methods to complete the fieldwork rather than the postponement of research projects any further. As the pandemic continues, institutions have taken a flexible approach to support their researchers and have advised to explore alternative methodology and methods to complete the research project and, where possible, explore virtual communication tools and use them to collect data for their research project. However, these virtual methods of data collection come up with its own challenges and opportunities which we have discussed below.

## USING VIRTUAL COMMUNICATION TOOLS FOR QUALITATIVE INTERVIEWS

The use of virtual technology is increasingly embedded in teaching and learning activities of students and has been widely used and appreciated by educators, technologists and students in every fields of the study for many years.^6–8^ Likewise, the use of virtual communication tools for data collection is not a new technique and it has been used for several decades while conducting survey.^9^ Sedgwick and Spiers has recommended that video conferencing is the most viable and cost-effective alternative to face-to-face in-depth interviewing to overcome geographical barriers and time constraints.^10^ Many researchers have commonly used Skype interviewing as data collection methods before,^11^ however researchers largely preferred face-to-face interviews before the pandemic. The restriction imposed due to COVID-19 has compelled researchers to explore virtual communication tools further and have provided an opportunity to grow knowledge and understanding about using information technology effectively in the research fieldwork. The popularity of Zoom, Microsoft Teams and Google Hangouts has become the saviour for the virtual conferences, research dissemination and networking during COVID-19. Similarly, the accessibility of voice/video calls through social media such as Facebook and WhatsApp have brought the connectivity to the masses, which is important if we want to use these virtual communication tools for qualitative interviews.

The use of virtual communication tools such as Zoom, Microsoft Teams, Google Hangouts, Facebook and WhatsApp have provided good alternatives for researchers to advance and progress with the data collection process to support their fieldwork. Our experiences of conducting interviews from research participants in both community settings and institutional settings of Nepal during COVID-19 pandemic showed that most participants were excited and felt safe to participate in virtual interviews. Also, it was easier to mutually agree a convenient time to conduct interviews and it has saved the time researchers would have spent travelling to conduct the fieldwork.

The first author, who is an international researcher doing her PhD at the UK university, was faced with the challenges of travelling to Nepal to collect the data for her research. In the current situation, where there are increasing number of COVID-19 cases in both Nepal and the UK, travelling was not an easy option and face-to-face interviews would have presented its own challenges as discussed earlier in this paper. Traditionally, if the researchers are not able to collect data themselves then they recruit research assistant(s), who is trained and managed by the researcher in line with the ethical guidelines, to facilitate the data collection process.^12,13^ However, the use of virtual communication tools has made it possible for the researchers to conduct the interviews themselves without recruiting research assistant(s), which is an advantage for the early career researchers in terms of gaining first-hand experience of interviewing participants. This enhance researchers experience of conducting fieldwork and provide opportunities to develop skills in conducting interviews.

The use of virtual communication tools has also allowed researchers to connect to the gatekeepers and equip them with ethical guidelines of autonomy, anonymity and confidentiality for the research during an initial preparation for the virtual data collection. Gatekeepers invariably play an important role in facilitating researchers' access to the participants^14^ and can play a crucial role in acting as a reliable person to provide post-interview safeguarding for the participants. However, researchers must make sure that the gatekeepers are not playing the role of research assistants and are not present during the interviews with the participants. There should be a clear understanding about the role and limitations of the gatekeepers among the researchers and those who are playing role of the gatekeepers.

## OVERCOMING CHALLENGES WHILE USING VIRTUAL COMMUNICATION TOOLS

Researchers have raised concerns that online interviews may limit the participation of people who do not have access to the internet connection or where the participants are going to incur charge for using the internet to participate in the interviews. In developing countries such as Nepal, it is important that the researcher do not expect the participants to incur any charge or costs for participating in the research. One way to tackle this issue is to provide the facility such as phone/tablet to the gatekeeper that can be kept at the interview site and can be used by all the participants. If the interview site is in a hospital, the internet connection can be freely accessible from the hospital WiFi network or the researcher should arrange the purchase of the internet data package to address this issue. Also, the researcher can purposefully plan to consider the location of the sampling unit where diverse participants are present for example, a hospital that covers a large number of people from different geographical location, wider sociocultural and economic background in the region. This strategy can address the issue of limitation of wider participation in the research. Lack of skills to use the technology in developing countries is still a challenge in the 21^st^ century.^15^ This means majority of people find it difficult to take part in virtual media interviews, and again it may limit the chances of approaching a wide range of participants in the research in the community settings. Therefore, the researcher must have an arrangement to address the issue and one of the options could be training the gatekeeper who can facilitate the technology. However, the researcher needs to make sure that the gatekeeper is not interfering or taking part in the interview process.

Many researchers are not convinced with the idea of virtual interviews, as PhD students and early career researchers would have the limitations to gain first-hand experience of the fieldwork for data collections and field observation. However, some researchers may argue that conducting a virtual fieldwork is much more challenging and provides the skills for future research, as the use of technology in the research is likely to increase post pandemic. The other challenges are when the research deals with sensitive topics or with vulnerable participants. For example, pregnant woman as a research participant may feel upset while sharing their experiences about mental health issues. However, it is important to highlight that video conferencing allows researcher to see the participants' facial expressions and body language, which allows researchers to make use of reflexivity in difficult circumstances.

For example, if the participant becomes uncomfortable during the interview, the researcher can give time and space that will help the participant to compose themselves.^11^ In addition, a reliable person such as gatekeepers, must be present or accessible at the interview site, but not in the same room for confidentiality purposes, to provide emotional support to the participants or signposting them to the available support and services upon the guidance of the researcher.

In our research fieldwork, we took a pre-determined 3-steps approach to the virtual conversations with the research participants i.e. rapport building, interview conversation and closing the conversation. Although this was not an in-person interviews, it was important for us to build rapport with the participant before discussing interview questions. This allowed us to neutralise the environment where participants felt more comfortable about discussing their life stories in relations to their health and wellbeing, which is important for any health related qualitative research. Following the interviews, it was important for us not to abruptly end the conversation, but to provide support by continuing with general conversations to protect the participant from vulnerability. Therefore, ending the conversation with information and message of support ensured that the participants left the conversation feeling strong and protected.

## WAYS FORWARD

In these unprecedented times of the COVID-19 pandemic, researchers should have a realistic plan for their research project rather than being too ambitious. As the pandemic continues, it is important that researchers are prepared with alternative methods for data collection. As the use of technology in learning and teaching has increase significantly, it is likely that this trend will be followed in the research fieldwork and dissemination process. Looking at the advantages of using virtual communication tools for conducting qualitative interviews, many researchers are likely to adopt this technique during and after the pandemic to overcome the time constraints and financial burden of the research process. If the challenges of the virtual communication tools are addressed appropriately, the virtual interviewing methods are likely to become a preferred choice rather than an alternate option.
